# First Assessment of Awareness and Knowledge of Glaucoma Among Citizens of Addu City, Maldives: A Cross-Sectional Study

**DOI:** 10.7759/cureus.44931

**Published:** 2023-09-08

**Authors:** Zainudheen Faroog, Saifudheen Faroog, Abdul R Zia Zaidi

**Affiliations:** 1 College of Medicine, Al Faisal University, Riyadh, SAU; 2 Family and Community Medicine, Alfaisal University, Riyadh, SAU

**Keywords:** sociodemographic factors, maldives, knowledge, awareness, glaucoma

## Abstract

Background

Glaucoma is a progressive optic neuropathy characterized by visual field loss and potentially irreversible blindness, which poses a significant global health challenge. The Maldives, renowned for its unique geography and vibrant culture, faces unique challenges in healthcare access and delivery due to its scattered islands. Limited access to specialized healthcare services, coupled with cultural and socioeconomic factors, can contribute to disparities in glaucoma awareness and early detection. Understanding glaucoma awareness is paramount due to its potential impact on public health and the healthcare system. The aim of this study is to assess the awareness and knowledge about glaucoma among the citizens of Addu City, Maldives.

Methods

A cross-sectional study was conducted among 390 residents of Addu City, Maldives. The online survey questionnaire assessed demographic information, knowledge and awareness of glaucoma, and risk factors associated with the disease.

Results

The majority of participants (66.9%) had never heard of glaucoma. Among those who had heard of glaucoma, only 22.6% knew that it could cause irreversible blindness. Logistic regression analysis and multivariate analysis showed that age, gender, socioeconomic status, and education level were significantly associated with awareness and knowledge of glaucoma (p<0.05).

Conclusion

Findings suggest a high likelihood of low awareness and knowledge of glaucoma among residents of Addu City, Maldives. The study highlights the urgent need for policymakers, program implementers, and the health sector to conduct awareness programs in the community and provide facilities for annual eye examinations, as well as to organize systematic screening plans for glaucoma across the Maldives.

## Introduction

Glaucoma, characterized by progressive optic neuropathy leading to visual field loss and potentially irreversible blindness, presents a significant global health challenge. The prevalent primary open-angle glaucoma and primary angle-closure glaucoma forms are often asymptomatic, making early recognition and treatment critical to prevent vision loss. It stands as a primary cause of avoidable blindness [1], negatively impacting an individual's socioeconomic well-being [2].

Statistically, glaucoma affects 3.54% of the global population aged 40 to 80 years [3]. In 2013, approximately 64.3 million individuals within this age range had glaucoma, projected to rise to 76.0 million by 2020 and 111.8 million by 2040 [3]. Notably, a meta-analysis of regional studies anticipates a 16.0% increase to 59.51 million cases in Asia by 2020 and a 57.6% rise to 80.87 million by 2040 [4]. Neighboring countries like India report over 11 million affected individuals [5].

The insidious nature of glaucoma, marked by early asymptomatic stages and the lack of reliable screening methods, contributes to its late-stage detection, with 50-90% of cases remaining undiagnosed [6,7]. Early diagnosis and treatment remain pivotal to avert glaucoma-induced blindness. Those at higher risk, such as people of Black ethnicity, first-degree relatives of glaucoma patients, and individuals above 40 years, should undergo annual eye screenings [7].

The public's engagement significantly influences the success of regular eye examinations. Awareness of the disease's nature and risk factors notably affects treatment-seeking behavior, particularly for asymptomatic conditions like glaucoma [8]. The level of glaucoma knowledge impacts its comprehension as an ocular ailment. Disparities between studies in different countries are attributed to urbanization, knowledge sources, education, and methodology [9].

Research in India demonstrates the association of glaucoma awareness with educational and socioeconomic status, showing higher awareness in individuals of higher socioeconomic groups and with higher education levels [10]. Similar trends are reported in Saudi Arabia, Nigeria, and Nepal [11-13], with some studies indicating gender or age-related correlations [14]. However, gender norms and behaviors are context-dependent [15].

In Saudi Arabia, friends/family constitute the primary information source on the disease [11]. A Syrian study echoed this trend, where friends were the main knowledge source for 32.2%, and hospitals, ophthalmologists' clinics, and health staff for 8% [16].

Previous studies in the Maldives, like the First Rapid Assessment of Avoidable Blindness Survey, did not include posterior segment evaluation through fundus photos or assess the retinal nerve fiber layer (RNFL) via optical coherence tomography (OCT), potentially missing retina, glaucoma, or optic nerve issues if visual acuity isn't concurrently reduced [17]. No specific literature exists on glaucoma awareness in the Maldives. Thus, gauging public awareness is pivotal for health programs aiming to enhance community knowledge of such conditions. Elevated awareness and knowledge are crucial for early diagnosis, management, and prevention of potential visual impairment.

## Materials and methods

Study design and setting

This was a population-based observational cross-sectional study conducted in Addu City, the Maldives' second-largest city, which has a population of 34,503 people, with 17233 men and 17270 women. The sample size was 385 respondents and was calculated using Rasoft (Raosoft Inc., Seattle, Washington) with the following parameters: target population size: 34,503 citizens, margin of error: 5%, confidence level: 95%, response Rate: 80%).

Study instrument

Survey questions and research methodology were adopted from previously published manuscripts that dealt with glaucoma awareness and knowledge in other populations [6, 15, 18]. Based on the review and considering the regional and local contexts, a self-administered questionnaire was developed. Both English and Dhivehi (local language of Maldives) versions of the survey questionnaire were prepared and pretested. Also, the questionnaire was reviewed by a biostatistician and by a group of experts in ophthalmology to ensure face validity and reliability.

Data collection

The data was collected from all six administrative areas of Addu City. A convenience sampling technique was used, and the data was collected by an online survey. All participants were explained about the objective and scope of this study along with consent, including voluntary participation. The inclusion criteria were all natives aged ≥18 years old residing in Addu City during data collection. The exclusion criteria were anyone who was under the age of 18 years and non-natives.

Statistical analysis

Completed questionnaires were analyzed using SPSS version 26 (IBM Inc., Armonk, New York). The categorical variables were presented as frequencies and percentages, whereas the numerical variables were presented as mean ± standard deviation. The association of awareness and knowledge about glaucoma and age, gender, and education level was compared using Chi-square, Independent t-test, one-way ANOVA, and Pearson correlation tests. A p-value of <0.05 was considered a statistically significant result.

Ethical approval and consent to participation

The study was approved by the National Health Research Council of Maldives (research registration No: NHRC/2022/25). The study complied with the principles of the Helsinki Declaration, ensuring that participation in the study was voluntary, and the names of the participants were not taken to ensure anonymity and confidentiality. Informed consent was obtained from the study participants prior to study commencement.

## Results

A total of 410 participants from various districts/islands in Addu City completed the survey. Twenty participants were rejected due to incomplete information and exclusion criteria. The age range of participants was 18-68, with a mean age of 39.04 ± 12.33 SD. Of the total participants, 273 (70%) were females and 117 (30%) were males (n=117). The most common levels of education were high school (102, 26.4%), secondary school (81, 20.8%), and bachelor's degree (58, 14.9%); 14.6% (N=57) did not go through any formal school system or did not attend any school. More than half of the participants, 62.3% (n=243), belonged to a middle socioeconomic stratum according to monthly income in the range of 5000-15000 Maldivian Rufiyaa (MVR). Table 1 presents the complete sociodemographic characteristics of the study participants.

**Table 1:** Sociodemographic characteristics of study participants

Variable		Frequency	Percentage
Gender	Female	273	70
	Male	117	30
Education level	Bachelor's degree	58	14.9
	High school	102	26.2
	Master's degree	32	8.2
	No schooling completed	57	14.6
	PhD or equivalent	5	1.3
	Primary	55	14.1
	Secondary School	81	20.8
Monthly income	Less than 5000 MVR	87	22.3
	5000-9,999 MVR	127	32.6
	10,000-15000 MVR	116	29.7
	more than 15,000 MVR	60	15.4
Have you been diagnosed with glaucoma?	Yes	5	1.3
	No	351	90
	I don't know	34	8.7
Any member of the family affected by glaucoma?	Yes	8	2.1
	No	243	62.3
	I don't know	139	35.6

Out of 390 participants, 261 (66.9%) were not aware of glaucoma, while 129 (33.1%) participants were aware of glaucoma. Moreover, when asked if a family has been affected by glaucoma, the majority of the participants answered no or don't know, 62.3%, and 35.6%, respectively. In addition, only 1.3% (n=5) of people were diagnosed with glaucoma, while the rest (98.7%) were not diagnosed with glaucoma.

A bivariate analysis was carried out using the Pearson correlation test to assess the linear relationship between participants' age and awareness of glaucoma. There was a significant relationship between participants' age and awareness of glaucoma (p=0.001). There was a small positive correlation between the two variables, and it was statistically significant (r=.17, p=0.001).

A one-way ANOVA was performed to compare the effect of educational level on the awareness of glaucoma; there was a statistically significant difference between groups as determined by one-way ANOVA (F(6, 383)=10.602, p<0.001). Since Levene's statistic is significant, the equal variance was not assumed. To check for individual differences between the groups, post hoc comparisons were assessed using Dunnett's T3 test, as shown in Table 2.

**Table 2:** Post hoc comparisons of awareness of glaucoma by education level (Dunnett's T3) p<0.05 is considered significant

Variable (education level)	Mean difference	Std error	p-value	95% CI interval
No schooling vs. secondary school	0.293	0.061	<0.001 *	0.10 – 0.48
No schooling vs. high school	0.291	0.056	<0.001 *	0.12 – 0.46
No schooling vs. bachelor's degree	0.499	0.072	<0.001 *	0.27 – 0.73
No schooling vs. master's degree	0.572	0.092	<0.001 *	0.28 – 0.87
Primary vs. bachelor's degree	0.406	0.081	<0.001 *	0.15 – 0.66
Primary vs. master's degree	0.480	0.099	<0.001 *	0.16 – 0.86
PhD vs. bachelor's degree	-0.048	0.254	1.000	-1.33 –1.23
PhD vs. master's degree	0.025	0.260	1.000	-1.23 –1.28

The test revealed the mean awareness for those who had no schooling completed (M=0.0526, SD=0.22528) was significantly lower than those who only had a secondary school education (M=0.3457, p<0.001), high school education (M 0.3431, p<0.001), bachelor's degree (M=0.5517, p<0.001) and master's degree (M=0.6250, p<0.001). Also, those who had primary level education completed (M=0.1544, SD=0.35581) were significantly different and lower from those who only had a bachelor's degree (M=0.5517, p<0.001) and master's degree (M=0.6250, p<0.001). However, no significant differences were detected between PhD holders and other levels of education.

Furthermore, there was a statistically significant difference between monthly income and awareness of glaucoma (F(3, 386)=20.633, p<0.001), as shown in Table 3. The test revealed the mean awareness for those who earned more than 15,000 per month (M=0.7167, SD=0.22528) was significantly higher than for those who earned 5000-9999 (M=0.1969, p<0.001), less than 5000 (M=0.2414, p<0.001), and those who earned 10,000-15,000 (M=0.3448, p<0.001). Chi-squared was done to assess the relationship between both diagnosed status and family history and awareness of glaucoma among the participants. Both being diagnosed with glaucoma (p=0.001) or having a family history of glaucoma (p≤0.001) had a significant relationship with the awareness of glaucoma, as shown in Table 4. No association was found between awareness of glaucoma and gender, with a p-value of 0.589.

**Table 3:** Relationship between diagnosed status, family history, and awareness of glaucoma p<0.05  is considered significant

Variable	Chi-squared	df	p-value
Diagnosed status	10.284^a^	1	0.001 *
Family history	16.524^a^	1	<0.001 *

**Table 4:** Post hoc comparisons of awareness of glaucoma by monthly income p<0.05 is considered significant

Monthly income (MVR)	Variable (other income)	Mean difference	Std error	p-value	95% CL interval
More than 15,000	Less than 5000 MVR	0.475	0.0746	<0.001 *	0.28 - 0.67
	5000 - 9999 MVR	0.520	0.0685	<0.001 *	0.34 - 0.70
	10,000 - 15,000 MVR	0.372	0.0735	<0.001 *	0.18 - 0.59

**Figure 1:**
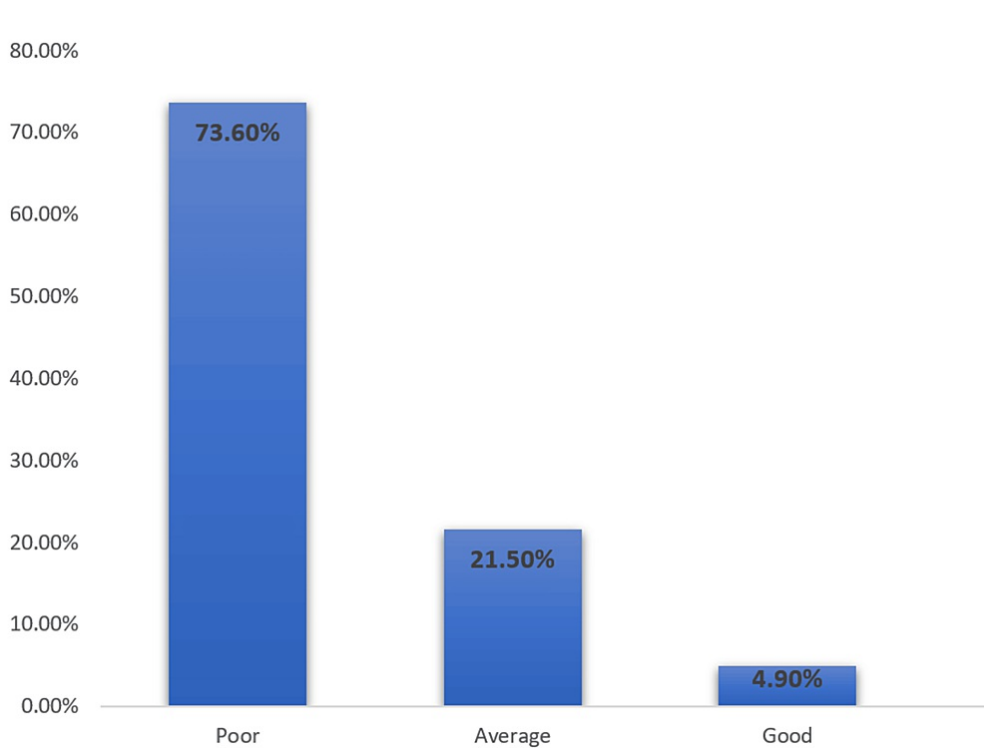
Participants knowledge level of glaucoma

The responses to a series of questions (10 questions) about glaucoma were used to evaluate the knowledge of glaucoma. Correct responses were coded as one, while incorrect responses were coded as zero. Three knowledge levels were used for people who scored according to the score obtained in the questionnaire (total score of 10): good (8 to 10 points), average (5 to 7 points), and poor (0 to 4 points). The mean test results for the knowledge of glaucoma were 2.7 out of 10. Only 4.9% (n=19) of participants had a good knowledge of glaucoma (their test results were from 8 to 10), while 21.5% (n=84) had average knowledge of glaucoma (test results were from 5 to 7). However, most of the respondents 73.6% (n=287) had poor knowledge (Figure 1). The knowledge results are detailed in Table 5 and Table 6.

**Table 5:** Knowledge of glaucoma among the residents of Maldives The correct answer has been marked with an asterisk ( * )

Question	Yes (%)	No (%)	I don't know (%)
The risk of glaucoma increases with age	22.8 *	25.4	51.8
Anyone can have glaucoma	31.5 *	21.5	46.9
Blindness from glaucoma can be prevented	29.2 *	14.6	56.2
Treatment of glaucoma is possible	51.3 *	10.5	38.2
Glaucoma has a familial predisposition	16.4 *	15.9	67.7
Glaucoma has an asymptomatic course	13.1 *	18.5	68.5
Vision is affected in the early course	16.7	18.7 *	64.6
Glaucoma is the same as cataract	8.5	45.6 *	45.9
What will happen in untreated glaucoma?	Slow, irreversible loss of vision 22.6% *	Eyes cannot be operated 25.4%	52.1

**Table 6:** Knowledge of glaucoma among the residents of Maldives The correct answer has been marked with an asterisk ( * )

Question	Progressive increase in glasses numbers	Pressure damage to the optic nerve	Mature cataract	Do not know
Glaucoma results from	16.4%	18.7% *	9.7%	55.1%

Regarding the specific knowledge of glaucoma (Table 5, 6), most of the respondents (202, 51.8%) were not aware that the risk of glaucoma increases with age, 219 (56.8%) did not know blindness from glaucoma can be prevented, 264 (67.7%) did not know that glaucoma has a familial predisposition, and 200 (51.3%) were aware that treatment of glaucoma is possible. More than half of the participants (68.5%, n=267) did not know that individuals could have glaucoma and were unaware of it (asymptomatic course), 215 (55.1%) did not know that glaucoma damages the optic nerve due to increased intraocular pressure. Also, 52.1% of the study population (n=203) displayed a lack of understanding that if glaucoma is untreated, it would progress over time with visual loss causing blindness.

There was a significant relationship between participants' age and awareness of glaucoma (p<0.001). There was a small negative correlation between the two variables, and it was statistically significant (r=-.26, p<0.001). There was a statistically significant difference between knowledge scores and education levels (F(6, 383)=11.97, p<0.001). The mean knowledge of glaucoma scores was statistically significantly higher in those who only had a master's degree (M=4.41, p<0.001), bachelor's degree (M=3.72, p<0.001), high school diploma (M=3.12, p<0.001), and secondary school education (M=2.63, p<0.001) compared to participants who never went to school with the lowest knowledge of glaucoma with a mean test of 0.86 (p<0.001).

Also, the knowledge of glaucoma scores was statistically significantly lower in those who only had a secondary school education (M=2.63, p<0.001) and primary school education (M=1.76, p<0.001) compared to participants who had a master's degree (M=4.41, p<0.001). Each participant's education group with their mean knowledge test results are shown in Table 7.

**Table 7:** The effect of education, monthly income, family history, medical history, and gender on the mean knowledge score of glaucoma p<0.05 is considered significant

Demographic factor	Variable	Mean glaucoma knowledge score	p-value
Education	No schooling completed	0.86	<0.001 *
	Primary school	1.76	
	Secondary school	2.63	
	High school	3.12	
	Bachelor's degree	3.72	
	Master's degree	4.41	
	PhD	3.80	
Monthly income	Less than 5000 MVR	2.28	<0.001 *
	5000 - 9999 MVR	2.13	
	10,000 - 15,000 MVR	2.52	
	More than 15,000 MVR	4.87	
Family history	Yes	5.75	0.004 *
	No	2.64	
	I don't know	2.63	
Diagnosed with glaucoma	Yes	5.40	0.034 *
	No	2.45	
Gender	Male	3.11	0.836
	Female	2.24	

There was a significant relationship between the monthly income of the participants and their knowledge of glaucoma (F(3,386)=19.01, p<0.001). Out of the 390 participants who answered this question, 60 participants earned more than 15,000 and had a mean knowledge of 4.87, 116 participants earned between 10k-15k and had a mean knowledge of 2.52, 127 participants earned between 5000 and 9999 had a mean knowledge of 2.13. Finally, 87 participants who earned less than 5k had a mean knowledge of 2.28, as shown in Table 7.

Family history (family members who were diagnosed with glaucoma) had a significant difference in the knowledge of glaucoma (F(2, 387)=5.70, p=0.004). Participants with a positive family history had a better knowledge of glaucoma than participants who had no family history and didn't know about their family history; the mean of the test results were 5.75, 2.64, and 2.63, respectively, as shown in Table 7. There was a significant difference between people who were diagnosed with glaucoma and their knowledge score (p=0.034). Five participants who had already been diagnosed with glaucoma had a mean knowledge of 5.4, and the rest of the 385 participants had a mean knowledge of 2.45, as shown in Table 7. In contrast, gender had no significant impact on the knowledge of glaucoma, with a p-value of 0.836.

The most common source of information about glaucoma for the participants was the internet, accounting for about 10% (39/390) of the participants; 9.2% were from family, relatives, and friends, and 6.9% were from social media. The rest of the results are shown in Table 8. However, 65.6% (n=256) had no source of information.

**Table 8:** Source of information for glaucoma awareness

Source	Percentage (%)
Friends/ family members	9.2
Healthcare provider (doctor/ nurse)	1.5
Internet	10
TV	2.3
Magazine	4.1
Social media	6.9
Radio	0.3
No source	65.6

## Discussion

The current study aimed to systematically evaluate the extent of awareness and comprehension concerning glaucoma among residents of Addu City, Maldives, employing a multiple-choice questionnaire specifically validated for this population. Notably, glaucoma is the second leading etiological factor for visual impairment in the adult global population, affecting an estimated 80 million individuals as of the year 2020. It is of particular interest that individuals of Asian descent constitute approximately 47% of this affected demographic [4].

The study discovered that only 33.1% of the study population in Maldives had an awareness of glaucoma. This is slightly lower than a study conducted in Damascus, Syria, where the percentage of awareness about glaucoma was 33.6% [16]. A study conducted in Abha, Saudi Arabia, showed a much higher awareness of 77.1% among the respondents [11]. Particularly, a recent study conducted within the southeast Asia region from central Nepal reported a higher level of awareness of glaucoma of 56.5% [13]. However, a higher awareness percentage was reported in a developing country such as Nigeria, which was 36.8% [18].

Despite the difference in the percentage of awareness across these countries, we cannot be convinced of the disparity for several reasons. One of the important factors to be considered is that both studies conducted in both Syria and Nepal were done in a hospital-based setting, and the study population was attending a tertiary care hospital. This difference has been seen in studies conducted in the same country with different populations. For example, a study conducted in tertiary care hospitals in central India reported glaucoma awareness of 27% [10], while awareness of glaucoma in rural populations of India ranges from 0.32% to 13.5% [6, 15, 19]. Another study done on patients attending an Ophthalmology Referral Center in New Mexico showed a higher awareness of 73.9% of the study population [20]. Therefore, we believe people attending health care settings, in particular eye departments, could have access to more information on common eye diseases, leading to higher awareness.

The study conducted in Nigeria with a higher glaucoma awareness could be explained by the fact that blindness due to glaucoma is highest in African countries [21], and many prevalence studies have already been conducted within the country [22, 23] and in other Sub-Saharan African countries to back this claim [24]. As a result of these prevalence studies, organizations such as WHO and UNICEF conducted awareness programs and provided screening facilities to prevent blindness from glaucoma. An example of this is a glaucoma consortium that was established in Ethiopia with the intent of raising public awareness about the disease in 2007 [25]. However, to this date, there are no population-based studies published on posterior segment eye diseases (PSEDs) to figure out the prevalence of glaucoma and exact blindness caused by this disease in the Maldives [17]. Moreover, the fact that the questionnaires are different, the population sample, and how the data were collected are further factors to be considered in these studies.

Overall, awareness in developing countries tends to be lower. A study conducted in rural areas in Bhaktapur, Nepal, reported a level of glaucoma awareness of 2.43% [26]. Nonetheless, studies conducted in developed countries such as Saudi Arabia and China reported higher awareness of 77.1% and 78.4%, respectively, among the respondents [11-27].

The huge disparity in awareness is explained by the difference in the country's Human Development Index (HDI), which constitutes life expectancy, adult literacy, and GDP per capita of a country. A high level of industrialization and per capita income is seen in developed countries while developing countries are at the early stages of industrial development and have a low per capita income. This enables developed countries to utilize their wealth and resources to fund the public's health care and health education. Even though the wealth and resources of a country depend on good governance of the leadership bodies, the government and healthcare workers can work simultaneously to improve health awareness in a country [15]. This highlights the necessity for our healthcare system and leadership to act accordingly to reduce the burden of glaucoma-related blindness in the upcoming future.

The results of this study highlight five important factors associated with the level of glaucoma awareness and knowledge in society. Firstly, our study found a significant relationship between age and awareness of glaucoma (r=.17, p=0.001). A similar relationship between age and awareness has been reported in studies conducted in both India and Nepal [10-13]. This can be explained by the fact that older age groups may have had more exposure or experience with glaucoma because it affects them more frequently in relation to the illness [13].

Although there was a significant relation between knowledge and age (p<0.001), it was a negative correlation (r=-0.21). Knowledge score was higher among the younger participants. This can be explained by the fact that younger participants were more likely to go through formal education and had more access to information resources such as the internet and social media.

Secondly, as predicted, education level remains a significant factor in awareness and knowledge. Respondents who were educated up to high school and above were more likely to be aware of glaucoma than those who only had primary or secondary education and those who had no formal schooling completed. This conclusion is similar to studies conducted in Saudi Arabia, India, and Syria, which found that respondents with a higher educational level were more likely to be aware and knowledgeable of glaucoma than those with a lower educational level [10, 11, 16]

Thirdly, glaucoma awareness and knowledge were high among respondents belonging to the upper socioeconomic class with an income above 15,000 compared to those who belonged to the lower SEC with an income less than 15,000. Studies done in India show a similar trend across society [15]. As a person belongs to a higher socioeconomic status, they are more likely to have broad and diverse relationships with professionals across various fields, including healthcare. Compared to their peers, this set of people (those who are high-income earners) have easier access to medical resources.

This is one of the first studies that explore the knowledge of various aspects of glaucoma. The findings of knowledge regarding glaucoma were also low in this study. The mean test results for the knowledge of glaucoma were 2.7 out of 10.

Although 45.9% of respondents were correct in knowing glaucoma is different from cataracts, 68.5% of people didn't know that glaucoma could be asymptomatic, and 52.1% didn't know about the irreversible nature of vision loss in glaucoma. Previous research has shown that most glaucoma patients go misdiagnosed, and many cases are discovered much later in the course of the disease due to the asymptomatic nature and arrive with a late stage of glaucoma after the eyes have sustained substantial and permanent damage [3,12]. Studies have shown that people who have heard about glaucoma and have some knowledge regarding the asymptomatic nature and irreversible blindness tend to get their eyes examined to measure the intraocular pressure [13, 28]. Hence, the primary goal of eye health campaigns should not only be to raise awareness but also to educate the public about the condition so that they will try to get a routine eye checkup every six months.

The main source of information for glaucoma was the internet. Other studies showed social relationships, such as having close relatives or friends diagnosed with glaucoma as their source of information [11, 16]. A study done in New Mexico reported people prefer to obtain information from an oral explanation or from an ophthalmologist [20]. However, as the world is moving towards globalization with many people having access to smartphones and the internet, utilization of social media such as Facebook, Instagram, and Twitter would be the fastest way to approach a large population within a short period of time. Also, educational videos can be run on television screens in the ophthalmology department or clinics while patients wait for their consultation, and brochures could be kept on the table for patients to read, thus enabling patients to ask questions to the doctor if they have any doubts or concerns. Additionally, World Glaucoma Day could be celebrated in hospitals and other public places where booths are organized for information sessions and free eye examinations for people over 40 years.

This is the first study conducted in Maldives to discuss awareness and knowledge of glaucoma. The limitations of the study must be taken into consideration when interpreting the results. First, it was conducted in a single city in the Maldives, and the results may not be generalizable to the entire Maldivian population. Larger multi-center studies across different geographical regions are needed to better understand glaucoma awareness and knowledge at the national level. Second, the study relied on a cross-sectional design, which precludes determining causality. Future longitudinal research could help identify factors influencing changes in awareness and knowledge over time. Third, data were collected via a self-administered questionnaire, which is subject to recall and social desirability biases. Participants may have over-reported their awareness and knowledge levels. Objective measures of glaucoma-related knowledge would strengthen the findings. Fourth, the convenience sampling technique used may have led to selection bias. Individuals who were not native residents or unavailable during data collection were excluded. A randomized population-based sampling approach is preferable. Fifth, awareness was assessed based on whether participants had heard of glaucoma previously, but deeper understanding was not evaluated. Future studies should use more comprehensive awareness assessment tools. Finally, no data were collected on glaucoma screening behaviors or risk factor assessment practices. Follow-up research could examine how awareness and knowledge relate to the uptake of eye care services for glaucoma detection and management. While this study provides valuable preliminary insight, additional large-scale longitudinal studies are needed to address its limitations and more robustly characterize glaucoma awareness in the Maldivian population.

## Conclusions

In conclusion, this cross-sectional study aimed to investigate the awareness and knowledge of glaucoma in the general population in Addu City, Maldives. The study found that the level of glaucoma awareness in Maldives was relatively low. The study highlights the need for healthcare systems and leadership to act accordingly to reduce the burden of glaucoma-related blindness in the future. The study also found a significant relationship between age and awareness of glaucoma, with older age groups being more aware of the disease. Education level also remained a significant factor in awareness and knowledge. The study results highlight the importance of public health education and awareness programs to improve knowledge and early detection of glaucoma. Further, population-based studies are needed in the Maldives to determine the prevalence of glaucoma and the exact burden of blindness caused by this disease.
